# Comparing the Incidence of Falls/Fractures in Parkinson’s Disease Patients in the US Population

**DOI:** 10.1371/journal.pone.0161689

**Published:** 2016-09-01

**Authors:** Linda Kalilani, Mahnaz Asgharnejad, Tuire Palokangas, Tracy Durgin

**Affiliations:** 1 UCB Pharma, Raleigh, North Carolina, United States of America; 2 UCB Pharma, Monheim, Germany; 3 UCB Pharma, Atlanta, Georgia, United States of America; UCSD School of Medicine, UNITED STATES

## Abstract

Patients with Parkinson’s disease (PD) may experience falls and/or fractures as a result of disease symptoms. There are limited data available from long-term studies estimating the incidence of falls/fractures in patients with PD. The objective was to compare the incidence rate of falls/fractures in PD patients with non-PD patients in a US population. This was a retrospective study using a US-based claims database (Truven Health MarketScan^®^) that compared the incidence rate of falls/fractures in PD subjects with non-PD subjects. The study period included the 12 months prior to index date (defined as earliest PD diagnosis [International Classification of Diseases, Ninth Revision, Clinical Modification code 332.0]) and a postindex period to the end of data availability. Fractures were defined by inpatient/outpatient claims as a principal or secondary diagnosis and accompanying procedure codes during the postindex period. Incidence rates and 95% CIs for falls/fractures were calculated as the number of events per 10,000 person-years of follow-up using negative binomial or Poisson regression models. Twenty-eight thousand two hundred and eighty PD subjects were matched to non-PD subjects for the analysis (mean [SD] age, 71.4 [11.8] years; 53% male). A higher incidence rate (adjusted for comorbidities and medications) of all fall/fracture cases and by fall and fracture types was observed for PD subjects versus non-PD subjects; the overall adjusted incidence rate ratio comparing PD to non-PD subjects was 2.05; 95% CI, 1.88–2.24. The incidence rate of falls/fractures was significantly higher in subjects with PD compared with non-PD subjects in a US population.

## Introduction

The incidence rate (IR) of Parkinson’s disease (PD), the second-most common neurodegenerative disorder, ranges between 1.5 and 22 per 100,000 person-years for all age groups, and up to 529 per 100,000 person-years in older populations [1,2]. Most patients with PD experience falls as a result of disease symptoms and in most cases have recurrent episodes. It is estimated that 60.5% of patients with PD experience at least one fall and 39% have recurrent falls [3]. The high frequency of falls consequently contributes to the increased risk for fractures in PD patients, which has been estimated to be approximately two times the risk in healthy controls [4]. It has been estimated that 76% of falls in PD patients require health care services and 33% result in fractures.[5] Falls and fractures may result in a series of unfavorable outcomes, such as disabilities and death.[6] Furthermore, among PD patients with fractures, the mortality rate is approximately 10.6% [6].

Despite the well-recognized increased risk in PD patients, the reported incidence of falls and fractures in PD patients have primarily been based on small studies, with estimates varying widely between 35% and 90% across studies [3]. Data are scarce on the incidence of falls and fractures in PD patients from large population-based studies with a long follow-up period [7]. Moreover, most of the available studies do not describe the age- or gender-specific incidence of falls and fractures.

With the prevalence of PD increasing, falls and fractures are expected to have a major impact on health care systems in the coming decades [8]. A better understanding of the IR of falls and fractures would be the first step to evaluate public health burden, assess individualized risk, develop effective interventions to reduce and manage these problems, and ultimately improve the quality of life in PD patients. To address these knowledge gaps, we conducted a retrospective cohort study using a large population-based claims data set in the United States, in which the IR of falls and fractures in matched populations of subjects with and without PD was evaluated and compared in terms of age, gender, and types of falls and fractures.

## Materials and Methods

### Data source

This retrospective cohort study was conducted using the Truven Health MarketScan^®^ Commercial Claims (CCMC [EV] 2012 v0.1), and the Truven Health MarketScan Medicare Supplemental and Coordination of Benefits (Medicare Supplemental; MDCR [EV] 2012 v0.1) databases. The CCMC database is composed of commercially insured individuals (ie, working-age adults and their dependents), whereas the MDCR database is composed of patients ≥65 years of age with Medicare coverage (a federal health insurance program) plus employer-paid commercial plans. The databases contain enrollment data, and medical and pharmacy claims and includes more than 100 million covered lives. Data are deidentified and comply with the patient confidentiality requirements of the Health Insurance Portability and Accountability Act.

### Study population

The study population was composed of a PD and comparator cohort of non-PD individuals. Subjects were eligible for the study if they were at least 40 years of age at the index date and were required to have a 12-month baseline period with continuous enrollment with medical and pharmacy coverage. The PD cohort was composed of subjects who had a PD diagnosis identified between January 1, 2008 and December 31, 2011. A PD diagnosis was defined as the presence of at least one inpatient claim with the International Classification of Diseases, Ninth Revision, Clinical Modification (ICD-9-CM) code 332.0 in any field of diagnosis, or at least two outpatient claims with the ICD-9-CM code 332.0 (occurring at least 30 days apart but within 365 days) in any field of diagnosis, or at least one outpatient claim with the ICD-9-CM code 332.0 in any field of diagnosis plus at least two prescription claims for PD-related medication (levodopa-carbidopa, anticholinergics, dopamine agonists, monoamine oxidase B inhibitors, or catechol-*O*-methyltransferase inhibitors), within 6 months of a PD diagnosis. Excluded from the analysis were subjects without any claim during the study period, or with a claims record of a diagnosis of secondary PD including drug-induced PD, vascular PD, essential tremor and dementia (ICD-9-CM code 332.1), dementia with Lewy bodies (ICD-9-CM code 331.82), metastatic cancer, osteogenesis imperfecta, bone cancer, lymphoma, leukemia, multiple myeloma, or Paget’s disease, in any field of diagnosis, at any time during the study period.

The comparator non-PD cohort was comprised of subjects without a claims record of a PD diagnosis at any time during the study period. These subjects were matched 1:1 within each database to the PD cohort by age (within 2 years), gender, region of residence of the patient, and duration of medical and pharmacy coverage in the pre- and postindex periods (exactly by month), and were required to have at least 1 recorded claim of a health encounter in the baseline period. The index date for the comparator cohort was matched to the calendar time of a subject in the PD cohort using the closest encounter claim to the index date of the matching case in the PD cohort.

### Study outcomes

Falls were captured by the presence of at least one inpatient or outpatient claim with the ICD-9-CM codes E880.x to E885.x and E888.x as a principal/first or second diagnosis. Falls under the ICD-9-CM E886.x code were excluded because we only included accidental and/or unexpected falls. The falls were classified into the following 3 mutually exclusive groups: (1) falls on the same level (E885.x), (2) falls on a different level (E880.x–E884.x), and (3) unspecified falls (E888.x). Fractures were captured by the presence of at least one inpatient or outpatient diagnosis claim with ICD-9-CM codes 800.xx to 829.xx and E887.x as a principal/first or second diagnosis with accompanying current procedural terminology codes for site-specific fracture repair procedures. We excluded subjects with pathologic fractures that may have resulted from conditions such as cancer, infection, osteomalacia, and Paget’s disease, identified using the ICD-9-CM code 733.1x. Subjects may have experienced more than one fracture during the follow-up period. A subsequent fracture was classified as a new fracture if it occurred at a different site of the body or if it occurred at the same site after more than 365 days following the first fracture [9]. Fractures were categorized by site into 1 the following categories: (1) hip fractures (ICD-9-CM code 820.xx), (2) pelvic fractures (ICD-9-CM code 808.xx), (3) femur fractures (ICD-9-CM code 821.xx), (4) vertebral fractures (ICD-9-CM codes 805.xx and 806.xx), and (5) upper limb fractures (humerus/radius/ulna fracture [ICD-9-CM codes 812.xx and 813.xx]).

### Other study measures

The prevalence of comorbidities associated with falls or fractures in the baseline period was captured by the presence of at least one inpatient claim or two outpatient claims (occurring at least 30 days apart but within 365 days) with the ICD-9-CM codes for each of the conditions in any field of diagnosis. The comorbidities included Alzheimer’s disease, dementia, vertigo, osteoporosis, multiple sclerosis, celiac disease, Crohn’s disease, ulcerative colitis, dementia, ankylosing spondylitis, depression, arthritis, hyperthyroidism, Cushing’s syndrome, hyperparathyroidism, chronic liver disease, vitamin D deficiency, malnutrition, impaired vision, orthostatic hypotension, mobility impairment related comorbidities, and postural instability. The Charlson Comorbidity Index (CCI) also was calculated to measure overall burden of disease [10,11]. In addition, the prevalence of medications known to be associated with falls or fractures, defined by the presence of at least three prescription claims of the medications in the baseline period, was also evaluated. These included osteoporosis treatment, androgen deprivation therapy, nonsteroidal anti-inflammatory drugs, cyclooxygenase-2 inhibitors, glucocorticoids (excluding nonsystemic forms such as topical or inhaled applications), enzyme-inducing anticonvulsants, thiazolidinediones, benzodiazepines, sedatives, digoxin, diuretics, and psychotropic drugs.

### Follow-up

The last date of enrollment in the postindex period for the PD patients was required to be earlier or on the date of the matched pair in the non-PD cohort. Subjects were followed up from the index dates to the first occurrence of any of the following: (1) date of last enrollment after the index date, for subjects who were not continuously enrolled through the end of the study period; (2) end date of the study period, for subjects who were continuously enrolled through the end of the study period; or (3) death.

### Statistical analysis

Descriptive demographic and clinical characteristics were presented as mean and standard deviation or median and interquartile range as appropriate for continuous variables, and comparisons between the cohorts were made using a paired *t* test. Categorical variables were presented as frequencies and percentages, and comparisons were made using the McNemar’s test. The IR of falls and/or fractures was calculated by dividing the total number of new cases of falls and/or fractures by the total number of person-years contributed by persons at risk in the time period of interest. For subjects with a subsequent fracture on the same site as the first fracture, the follow-up time was calculated up to the first fracture and follow-up resumed 365 days after the first fracture. For subjects who had subsequent fractures at a different site from the first fracture, follow-up time was continuously calculated. In subjects who experienced fractures at multiple sites, the fractures were counted as 1 episode for the overall IR estimation if the ICD-9-CM codes for the fractures were generated within a period of 7 days. For fracture site-specific IRs, the fractures were counted separately.

Crude and adjusted IRs and incidence rate ratios (IRRs) were computed using the negative binomial model. However, if the negative binomial model did not converge, the analysis was done using Poisson regression model. All the matching variables (age, gender, region of residence, and duration of enrollment) were included in the adjusted models. Confounding of the other factors was assessed using the change in estimate criteria, with variables causing an absolute change in estimate of at least 0.10 considered confounders and retained in the final model. A backward elimination strategy was used to select the final model. The goodness of fit of the final model was assessed using the deviance test and examining the distribution of standardized Pearson residuals for covariate patterns that were outliers. Sensitivity analyses were performed by varying the restricting the cases of PD to the principal diagnosis. All statistical tests were 2 sided and a *P*<0.05 value was considered significant. The analysis was conducted using SAS version 9.3 software (SAS Institute, Inc., Cary, NC, USA).

## Results

### Study population characteristics

We identified 28,280 eligible PD subjects and 28,280 matched non-PD subjects with same distribution of the matched factors. Both cohorts comprised 53.3% males. The mean age was 71.4 years (SD, 11.82) and 67.4% of the subjects were aged at least 65 years. The geographic distribution of the study population was as expected by the geographic representation in the Truven Health MarketScan Research Databases, with 68% of the subjects from the South and North Central regions. Most of the subjects were receiving supplemental Medicare insurance. The prevalence of baseline medications and comorbidities in the PD and non-PD comparator cohorts are shown in **Table 1**. The PD subjects were more likely to use medications associated with falls and fractures, and generally had a higher prevalence of comorbidities than their non-PD comparators.

**Table 1:** Baseline characteristics of study participants.

Characteristic, n (%)	All, n = 56,560	PD cohort n = 28,280	Non-PD cohort n = 28,280	*P* value^a^
Age, y				
40–54	4882 (8.6)	2441 (8.6)	2441 (8.6)	
55–64	13575 (24.0)	6789 (24.0)	6786 (24.0)	
≥65	38103 (67.4)	19050 (67.4)	19053 (67.4)	
Gender				
Female	26410 (46.7)	13205 (46.7)	13205 (46.7)	
Male	30150 (53.3)	15075 (53.3)	15075 (53.3)	
Payer type				<0.0001
Commercial	17636 (31.2)	9040 (32.0)	8596 (30.4)	
Medicare	38924 (68.8)	19240 (68.0)	19684 (69.6)	
Medications^b^				
Osteoporosis treatment	5385 (9.5)	2602 (9.2)	2783 (9.8)	0.0095
Androgen deprivation therapy	2002 (3.5)	907 (3.2)	1095 (3.9)	<0.0001
NSAIDs	4336 (7.7)	1931 (6.8)	2405 (8.5)	<0.0001
Glucocorticoids	1949 (3.4)	826 (2.9)	1123 (4.0)	<0.0001
Thiazolidinediones	2275 (4.0)	812 (2.9)	1463 (5.2)	<0.0001
Benzodiazepines	6937 (12.3)	4103 (14.5)	2834 (10.0)	<0.0001
Sedatives	2715 (4.8)	1266 (4.5)	1449 (5.1)	0.0003
Digoxin	2002 (3.5)	896 (3.2)	1106 (3.9)	<0.0001
Diuretics	16638 (29.4)	6778 (24.0)	9860 (34.9)	<0.0001
Psychotropic drugs	12403 (21.9)	7960 (28.1)	4443 (15.7)	<0.0001
CCI Comorbidity Index				
≥4	1955 (3.5)	1226 (4.3)	729 (2.6)	<0.0001
Other comorbidities^b^				
Osteoporosis	1016 (1.8)	558 (2.0)	458 (1.6)	0.0015
Multiple sclerosis	144 (0.3)	93 (0.3)	51 (0.2)	0.0005
Depression	2829 (5.0)	1974 (7.0)	855 (3.0)	<0.0001
Arthritis	6014 (10.6)	3192 (11.3)	2822 (10.0)	<0.0001
Malnutrition	340 (0.6)	250 (0.9)	90 (0.3)	<0.0001
Impaired vision	5861 (10.4)	2515 (8.9)	3346 (11.8)	<0.0001
Orthostatic hypotension	869 (1.5)	585 (2.1)	284 (1.0)	<0.0001
Postural instability	2378 (4.2)	1986 (7.0)	392 (1.4)	<0.0001
Alzheimer’s disease	1210 (2.1)	931 (3.3)	279 (1.0)	<0.0001
Dementia	1361 (2.4)	1032 (3.6)	329 (1.2)	<0.0001
Vertigo	1464 (2.6)	918 (3.2)	546 (1.9)	<0.0001
Mobility impairment-related comorbidities	3414 (6.0)	2772 (9.8)	642 (2.3)	<0.0001

CCI, Charlson Comorbidity Index; NSAID, nonsteroidal anti-inflammatory drug; PD, Parkinson’s disease.

^a^*P* values were based on McNemars test

^b^The counts and percentages in patients not using the medication or without the comorbidity were not shown.

### IR and IRR of Falls or Fractures

During the follow-up period, we identified a total of 2063 and 1014 subjects who experienced falls or fractures in the PD and non-PD cohorts, respectively. As shown in **Table 2**, the overall IR of falls/fractures was significantly higher in the PD cohort than in the non-PD cohort, in the crude analysis (IRR, 2.08; 95% CI, 1.90–2.27), and after adjusting for confounders (adjusted IRR, 2.05; 95% CI, 1.88–2.24). The increased risk of falls in patients with PD subjects was observed across all age groups assessed, even after adjusting for confounders (40–54 years: IRR, 1.83; 95% CI, 1.21–2.78; 55–64 years: IRR, 2.25; 95% CI, 1.79–2.83; ≥65 years: IRR, 2.03; 95% CI, 1.84–2.24). Males had a slightly higher relative risk than females when the PD and non-PD cohorts were compared (females: IRR, 1.96; 95% CI, 1.75–2.18; males: IRR, 2.23; 95% CI, 1.93–2.59).

**Table 2:** IRs and IRRS of falls or fractures, overall and by age and gender.

	PD (n = 28,275)			Non-PD (n = 28,275)			Crude IRR (95% CI)	Adjust IRR^a^ (95% CI)
	Cases	Person-years	Adjusted IR (95% CI)^a^^,^^b^	Cases	Person-years	Adjusted IR (95% CI)^a^^,^^b^		
Total	2,063	59,922	342.7 (323.0, 363.6)	1,014	57,922	166.9 (154.8, 180.0)	2.08 (1.90, 2.27)	2.05 (1.88, 2.24)
Age (years)								
40–54	78	5,611	128.6 (96.1, 172.0)	44	5,612	70.2 (48.9, 101.0)	1.82 (1.21, 2.76)	1.83 (1.21, 2.78)
55–64	301	13,995	207.2 (178.2, 240.9)	136	13,991	92.1 (75.4, 112.5)	2.25 (1.78, 2.84)	2.25 (1.79, 2.83)
≥65	1,684	38,316	453.8 (425.5, 484.0)	834	38,319	223.2 (205.6, 242.2)	2.06 (1.86, 2.66)	2.03 (1.84, 2.24)
Gender								
Male	707	30,586	243.0 (221.1, 267.1)	321	30,568	108.7 (95.8, 123.4)	2.29 (1.97. 2.66)	2.23 (1.93, 2.59)
Female	1,356	27,336	509.6 (473.7, 548.2)	747	27,336	260.3 (237.6, 285.1)	1.97 (1.76, 2.20)	1.96 (1.75, 2.18)

^a^ Adjusted for age, gender, region of residence, and duration of enrollment.

^b^ Incidence rate is per 10,000 person-years.

Abbreviations: IR, incidence rate; IRR, incidence rate ratio; PD, Parkinson's disease.

### IR and IRR of Falls

**Fig 1:**
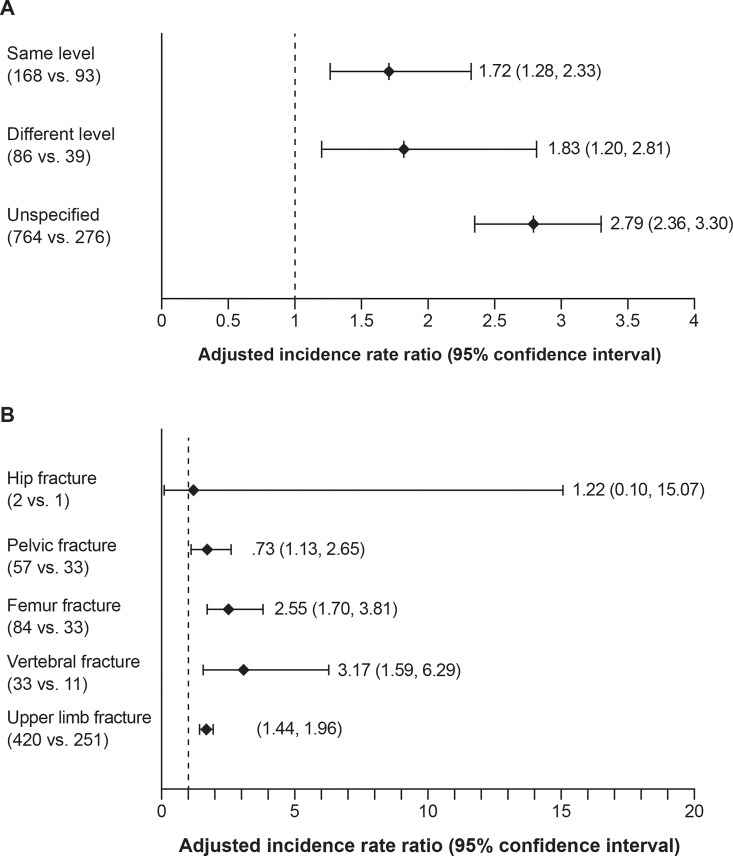
Adjusted incidence rate ratios of by types of falls and fractures. * (A) Adjusted incidence rate ratios by types of falls. (B) Adjusted incidence rate ratios by types of fractures. *The incidence rate ratios were adjusted for age, gender, region of residence, and duration of enrollment. Numbers in the parentheses under each type of falls/fractures were numbers of cases in the PD and non-PD cohorts, respectively. PD, Parkinson’s disease.

A total of 1018 falls in the PD cohort and 408 falls in the non-PD cohort were identified during the follow-up period. In approximately 73% of the cases, the type of fall was not specified (PD, n = 764; non-PD, n = 276). Among the falls in which the type was specified, the same-level falls were more common than different-level falls in both cohorts. The adjusted IR of same-level falls was 27.2 per 10,000 person-years (95% CI, 22.1–33.5) in the PD cohort and 15.8 per 10,000 person-years in the non-PD cohort (95% CI = 12.3, 20.3), respectively, whereas for different-level falls, it was 11.3 per 10,000 person-years (95% CI, 8.3–15.2) in the PD cohort and 6.1 per 10,000 person-years (95% CI, 4.2–8.9), respectively. The risk of falls was significantly higher in PD cohort than non-PD cohort for all types of falls, particularly for unspecified-level falls (**Fig 1A**).

### IR and IRR of Fractures

There were 1073 and 622 fractures identified during the follow-up period in the PD cohort and non-PD cohort, respectively. As shown in **Table 3**, the adjusted IR of fractures was 164.5 per 10,000 person-years (95% CI, 152.7–177.3) in the PD cohort, higher than the 95.3 per 10,000 person-years (95% CI, 87.0–104.3) in the non-PD cohort. The increased risk for fractures in PD subjects remained after adjusting for confounders (adjusted IRR, 1.73; 95% CI, 1.56–1.91), and was observed in all age groups assessed (40–54 years: adjusted IRR, 1.50; 95% CI, 0.94–2.39; 55–64 years: adjusted IRR, 1.91; 95% CI, 1.49–2.47; and ≥65 years: adjusted IRR, 1.71; 95% CI, 1.52–1.91). The PD-associated higher risk for fractures was also seen in both females (adjusted IRR, 1.70; 95% CI, 1.51–1.92) and males (adjusted IRR, 1.78; 95% CI, 1.48–2.15).

**Table 3:** Incidence rates and rates of ratios of fractures, overall and by age and gender.^a^

	PD (n = 28,275)			Non-PD (n = 28,275)			Crude IRR (95% CI)	Adjust IRR^b^ (95% CI)
	Cases	Person-years	Adjusted IR^b^^,^^c^ (95% CI)	Cases	Person -years	Adjusted IR^b^^,^^c^ (95% CI)		
Total	1,073	57,922	164.5 (152.7, 177.3)	622	57,922	95.3 (87.0, 104.3)	1.73 (1.56, 1.91)	1.73 (1.56, 1.91)
Age (years)								
40–54	46	5,611	72.6 (51.0, 103.4)	31	5,612	48.4 (32.1, 73.0)	1.48 (0.94, 2.34)	1.50 (0.94, 2.39)
55–64	178	13,995	120.0 (100.6, 143.2)	93	13,991	62.7 (50.0, 78.6)	1.92 (1.48, 2.48)	1.91 (1.49, 2.47)
≥65	849	38,316	202.9 (186.7, 220.4)	498	38,319	118.9 (107.5, 131.5)	1.71 (1.52, 1.91)	1.71 (1.52, 1.91)
Gender								
Male	321	30,586	106.5 (93.8, 120.9)	180	30,568	59.7 (50.8, 70.0)	1.78 (1.49, 2.14)	1.78 (1.48, 2.15)
Female	752	27,336	272.0 (249.9, 296.1)	442	27,336	159.7 (143.9, 177.2)	1.70 (1.51, 1.91)	1.70 (1.51, 1.92)

^a^ Includes fractures from all sites of the body.

^b^ Adjusted for age, gender, region of residence, and duration of enrollment.

^c^ Incidence rate is per 10,000 person-years.

Abbreviations: IR, incidence rate; IRR, incidence rate ratio; PD, Parkinson's disease.

When stratified by fracture site, upper limb fractures were the most common type of fracture, making up approximately 40% of all identified fractures in both cohorts (PD, n = 420; non-PD, n = 251). The IR of upper limb fractures was the highest in both the PD (adjusted IR, 57.8 per 10,000 person-years; 95% CI, 51.1–65.5) and non-PD cohorts (adjusted IR, 34.4 per 10,000 person-years; 95% CI, 29.7–39.9), followed by femur fractures (PD: adjusted IR, 12.3 per 10,000 person-years; 95% CI, 9.3–16.4; non-PD adjusted IR, 4.8 per 10,000 person-years; 95% CI, 3.3–7.1). The PD cohort had a significantly higher risk of fractures for sites assessed (pelvic fractures: adjusted IRR, 1.73; 95% CI, 1.13–2.65; femur fractures: adjusted IRR, 2.55; 95% CI, 1.70–3.81; vertebral fractures: adjusted IRR, 3.17; 95% CI, 1.59–6.29; upper limb fractures: adjusted IRR, 1.68; 95% CI, 1.44–1.96; **Fig 1B**).

## Discussion

By using the large population-based claims data from the Truven Health databases, this retrospective cohort study found that PD subjects were almost twice as likely to have falls and fractures as non-PD subjects, independent of age and gender. Moreover, the subgroup analysis showed that the increased risk in PD subjects was particularly high for unspecified falls and vertebral fractures. The incidence of falls and fractures increased with age in both the PD and non-PD cohorts. In the non-PD cohort, the risk of falls and fractures was relatively stable before 65 years of age, but doubled after 65 years of age, whereas in PD patients the risk rose steadily from 40 years of age. This difference has also been observed in previous studies [6], and indicates that factors intrinsic to PD such as disease severity and functional impairment might be more important determinants of the risk of falls and fractures in PD patients.

Our results confirmed those of other studies showing that females had a higher incidence of both falls and fractures in both the general population and PD patients. [4,12]. This gender disparity may be a result of menopause-associated osteoporosis [13,14]. Interestingly, in this study, the relative risk of falls and fractures between male PD subjects and male non-PD comparators (IRR, 2.23; 95% CI, 1.93–2.59) was more pronounced than that between female PD subjects and female non-PD comparators (IRR, 1.96; 95% CI, 1.75–2.18). Similar results also were observed in previous studies [6,15,16]. The excess relative risk in male PD compared with female PD subjects has not been assessed in previous studies, and consequently has previously received little attention. Male PD patients may have a different pattern of activities from female PD patients, perhaps having more desire to be mobile and consequently increasing the risk for falls and fractures.

The estimate of the association between PD and fractures in our study (IRR, 1.73; 95% CI, 1.56–1.91) is lower than what has been reported in previous studies. In a large nationwide population-based claims study of people aged 40 years and older living in Taiwan, the estimate of the risk for fractures in PD patients was slightly higher (adjusted hazard ratio, 2.25; 95% CI, 1.97–2.58) [6]. A meta-analysis using pooled data from 9 studies also reported a slightly higher risk in PD patients compared with non-PD patients (odds ratio, 2.28; 95% CI, 1.83–2.83) than what we have found [4]. It is well recognized that the risk of falls and fractures increases with disease severity and duration [17–19]. Our study includes both prevalent and incident cases of PD, which may have contributed to lower estimates compared with those reported in other studies, in addition to the differences in the study geographic location, patient demographics, and follow-up periods.

Hip fractures have been reported as the most common site of fractures and have shown the strongest association with PD in some previous studies [16,20,21]. In this study, upper limb fractures were the most common type of fracture experienced in both the PD and non-PD cohorts, and vertebral fractures demonstrated the strongest association with PD. Few cases of hip fractures were identified in this study. The risk of fractures by sites is determined by many factors. For example, osteoporosis and low body fat/body mass index has been shown to be associated with increased risk of hip fractures [22–24], and low bone mineral density particularly affects the risk of fracture for the hip, wrist, and spine [4]. Other factors include postural hypotension, dementia, and medications. The variation in the distribution of these factors might explain the distribution of fractures by sites across studies. Moreover, fracture misclassification (such as hip and femur fractures) also might contribute to the inconsistency in incidence rates of fractures by site [25].

Fall types could provide information regarding the causes of falls (eg, slips, trips, or falls from chair or bed); however, limited data are available so far. Based on an analysis of fall-related hospital admissions in elderly Australian patients (≥65 years of age), the ratio of same-level falls to different-level falls, respectively, was 2.1 (35.8% vs 16.9%) [26], which is a ratio similar to what we found (PD cohort: ratio, 1.96; 16.5% vs 8.4%; non-PD cohort: ratio, 2.4; 22.7% vs 9.6%). When comparing PD and non-PD subjects aged 65 years and older, we also found that PD patients were more likely to have same-level falls (ratio, 3.0) compared with non-PD subjects (ratio, 1.7). This difference demonstrates that patients with PD might be aware of falling risk when changing level, but may be less vigilant in regular daily activities.

There are limitations for this study based on the type of claims data. Firstly, the study population is limited to patients with commercial or Medicare supplemental insurance. Consequently, the subjects may not represent the general US population. Secondly, there might be missing information, miscoding, or underreporting of disorders in the claims data. However, these issues likely affect equally between the PD and non-PD cohorts without biasing our estimation substantially. Thirdly, although the accuracy of major diagnosis codes from the claims database has been considered acceptable, validity of the PD, falls, fracture, and comorbidity codes might still be a limitation of this study. Lastly, previous studies showed that the risk of falls and fractures varied by PD duration, severity, body composition, and psychological factors (eg, depression) [17,27]. We lacked this information to evaluate their influence on the incidence and PD-associated risk of falls/fractures.

## Conclusion

In conclusion, our study provides reliable estimates of the IR of falls and fractures in PD patients, and shows that patients with PD are at an increased risk for falls and fractures, especially unspecified falls and upper limb fractures. Our findings help identify the subpopulation in need, may inform clinical practice, and can assist in the development of guidelines for disease management in PD patients. Future studies to understand the incidence and risk of falls and fractures by PD stage and duration, and the impact of anti-PD drugs on these estimates are needed to further guide treatment strategies with an aim in reducing morbidity and mortality in this fragile population.
